# α2AP is associated with the development of lupus nephritis through the regulation of plasmin inhibition and inflammatory responses

**DOI:** 10.1002/iid3.302

**Published:** 2020-03-31

**Authors:** Yosuke Kanno, Mei Miyashita, Mariko Seishima, Osamu Matsuo

**Affiliations:** ^1^ Department of Clinical Pathological Biochemistry, Faculty of Pharmaceutical Science Doshisha Women's College of Liberal Arts Kyoto Japan; ^2^ Department of Dermatology Gifu University Graduate School of Medicine Gifu Japan; ^3^ Kindai University Faculty of Medicine Osakasayama Japan

**Keywords:** Alpha2‐antiplasmin, inflammation, lupus nephritis

## Abstract

**Introduction:**

Lupus nephritis (LN) is a common complication of systemic lupus erythematosus (SLE), which is a chronic autoimmune disease. However, the detailed mechanisms underlying this disorder have remained unclear. Alpha2‐antiplasmin (α2AP) is known to perform various functions, such as plasmin inhibition and cytokine production, and to be associated with immune and inflammatory responses.

**Methods:**

We investigated the roles of α2AP in the pathogenesis of LN using a pristane‐induced lupus mouse model.

**Results:**

The levels of plasmin‐α2AP complex and α2AP were elevated in the lupus model mice. In addition, α2AP deficiency attenuated the pristane‐induced glomerular cell proliferation, mesangial matrix expansion, collagen production, fibrin deposition, immunoglobulin G deposition, and proinflammatory cytokine production in the model mice. We also showed that interferon‐γ (IFN‐γ), which is an essential inducer of LN, induced α2AP production through the c‐Jun N‐terminal kinase (JNK) pathway in fibroblasts. In addition, plasmin attenuated the IFN‐γ‐induced proinflammatory cytokine production through the AMPK pathway in macrophages, and α2AP eliminated these effects. Furthermore, we showed that α2AP induced proinflammatory cytokine production through the ERK1/2 and JNK pathways in macrophages.

**Conclusion:**

α2AP regulates the inflammatory responses through plasmin inhibition and proinflammatory cytokine production and is associated with the development of LN. Our findings may be used to develop a novel therapeutic approach for SLE.

## Graphical abstract

Alpha2‐antiplasmin (α2AP) regulates the inflammatory responses through plasmin inhibition and proinflammatory cytokine production and is associated with the development of lupus nephritis (LN).

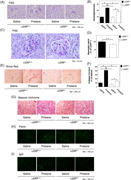

## 1 INTRODUCTION

Systemic lupus erythematosus (SLE) is an autoimmune disorder that affects multiple organ systems including the kidney, skin, lung, and heart.^1^ Lupus nephritis (LN) is a major cause of morbidity and mortality that affects more than half of patients with SLE during the disease progression.^2^, ^3^ Persistent inflammation caused by LN induces the activation of mesangial cells, endothelial‐to‐mesenchymal transition, infiltration of cellular mediators, and causes progressive glomerulosclerosis and tubulointerstitial fibrosis.^2^, ^4^ It has been reported that autoantibody production, immune complex deposition, hypercoagulability, and thrombosis are associated with LN progression.^5^, ^6^, ^7^, ^8^ However, the precise mechanisms of LN pathogenesis have remained unclear.

Alpha2‐antiplasmin (α2AP), which is synthesized in various tissues including kidney and liver, rapidly inhibits plasmin, a main enzyme of the fibrinolytic system, and results in the formation of a stable inactive complex, plasmin‐α2AP complex (PAP).^9^, ^10^, ^11^ On the other hand, α2AP is known to have various functions, such as cytokine production, cell growth, cell differentiation, and to be associated with angiogenesis, immune and inflammatory responses, tissue remodeling, fibrosis, brain functions, and bone formation.^12^, ^13^, ^14^, ^15^, ^16^, ^17^, ^18^, ^19^ The increased circulating levels of PAP and α2AP, and the impaired fibrinolysis have been reported in patients with SLE.^7^, ^8^ These findings suggest that α2AP may play a critical role in the development of SLE.

The administration of pristane, a mineral oil (TMPD), has been reported to induce severe kidney disorder, characterized by glomerular cell proliferation, mesangial matrix expansion and inflammation, and cause lupus‐like disease.^20^, ^21^ In the present study, we examined the role of α2AP in the pathogenesis of LN using a pristane‐induced lupus mouse model, and demonstrated that α2AP is associated with the development of LN through the regulation of plasmin inhibition and inflammatory responses.

## 2 MATERIALS AND METHODS

### 2.1 Animals

The α2AP deficient (α2AP^−/−^) mice were generated by homologous recombination using embryonic stem cells, as described previously.^22^ Wild type (α2AP^+/+^) and α2AP^−/−^ mice littermates were housed in groups of two to five in filter‐top cages with a fixed 12‐hour light and 12‐hour dark cycle. The animal experiments in this study were approved by the Animal Research Committee of Doshisha Women's College of Liberal Arts (approval ID: Y16‐013, Y17‐013).

### 2.2 Mice experiments

The saline or pristane (500 μl/body; Funakoshi; Tokyo, Japan) were administered intraperitoneally once in male mice for 3 months. The samples of kidney were placed immediately in liquid nitrogen, and stored at −80°C until use.

### 2.3 Enzyme‐linked immunosorbent assay

The serum levels of PAP and anti‐dsDNA antibody in mice were measured by mouse PAP enzyme‐linked immunosorbent assay (ELISA) kit (Elabscience, TX) and anti‐dsDNA antibody mouse ELISA kit (Shibayagi, Gunma, Japan), respectively. The absorbance of the ELISA samples was measured at 450 nm using an EL 340 Bio Kinetic Reader (Bio‐Tek Instruments Inc, VT).

### 2.4 Glomerular cell proliferation and mesangial matrix expansion

The glomerular cell proliferation and mesangial matrix expansion were assessed using periodic acid Schiff (PAS) staining and masson trichrome staining. The stained images obtained from separate fields on the specimens were analyzed by using ImageJ.

### 2.5 Collagen content in kidney

The collagen content was measured as previously described.^23^ The collagen content was assessed using sirius red staining. In these assays, sections are stained with sirius red as described by Junqueira et al.^24^ After deparaffinization, the sections are treated in 0.2% phosphomolybdic acid for 5 minutes. Next, the section stained in 0.1% sirius red for 90 minutes and 0.01 N HCl for 2 minutes. The stained images obtained from separate fields on the specimens were analyzed by using ImageJ. Sirius red positive area was expressed as a percent of the observed with sham mice.

### 2.6 Immunohistochemical staining

We performed immunohistochemical staining as previously described.^25^ Paraffin sections were labeled with each antibody, then labeled with fluorescein isothiocyanate‐conjugated immunoglobulin G (IgG; Thermo Fisher Scientific, CA). The signals in the kidney section were then detected using a laser‐scanning microscope.

### 2.7 Western blot analysis

We performed Western blot analysis as previously described.^26^ The kidney samples from mice were homogenized and sonicated in the lysis buffer. The protein concentration in each lysate was measured using a BCA Protein Assay Kit (Pierce, IL). Proteins in the supernatant were separated by electrophoresis on 10% sodium dodecyl sulfate–polyacrylamide gels and transferred to a polyvinylidene fluoride membrane. We detected each protein by incubation with each antibody followed by incubation with horseradish peroxidase‐conjugated antibodies to IgG (Santa Cruz Biotechnology, CA).

### 2.8 Cell culture

NIH3T3 fibroblasts and RAW 264.7 macrophages were seeded into the 60‐mm diameter dishes and maintained in 2 mL Dulbecco's modified Eagle's medium (DMEM) containing 10% fetal calf serum at 37°C in a humidified atmosphere with 5% CO_2_/95% air. The medium was replaced with serum‐free DMEM. Then, the cells were used for experiments.

### 2.9 Statistical analysis

All data were expressed as mean ± SEM. The statistical analysis was conducted with unpaired *t*‐test for two‐group comparison, with one‐way analysis of variance followed by the least significant difference test for multiple comparisons. Statistical significance was defined as *P*  < .05.

## 3 RESULTS

### 3.1 **The levels of** plasmin‐α2AP complex **and** alpha2‐antiplasmin **were elevated in the pristane‐induced lupus mouse model**

**Figure 1:**
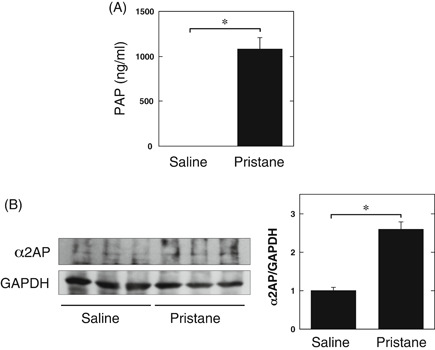
The levels of plasmin‐α2AP complex (PAP) or alpha2‐antiplasmin (α2AP) in the pristane‐induced lupus mouse model. A, The levels of PAP in the serum from saline or pristane‐treated mice was determined by enzyme‐linked immunosorbent assay (saline: n = 4, pristane: n = 3). B, The expression of α2AP in kidney from the saline or pristane‐treated mice was examined by Western blot analysis. The histogram shows quantitative representations of α2AP (n = 3). The data represent the mean ± SEM. *P < .01

The administration of pristane is known to induce LN, which is associated with the development of glomerular cell proliferation, mesangial matrix expansion, and inflammation.^20^, ^21^ The levels of PAP in the serum from pristane‐induced lupus mouse model were assessed by ELISA. The levels of PAP in the serum from pristane‐treated mice were significantly higher in comparison to saline‐treated mice (Figure 1A). We also examined the expression of α2AP in the kidney from pristane‐induced lupus mouse model by a Western blot analysis. The expression of α2AP in the kidney from pristane‐treated mice was significantly higher than that in saline‐treated mice (Figure 1B).

### 3.2 **α2AP deficiency attenuated the development of** lupus nephritis **in the pristane‐induced lupus mouse model**

**Figure 2:**
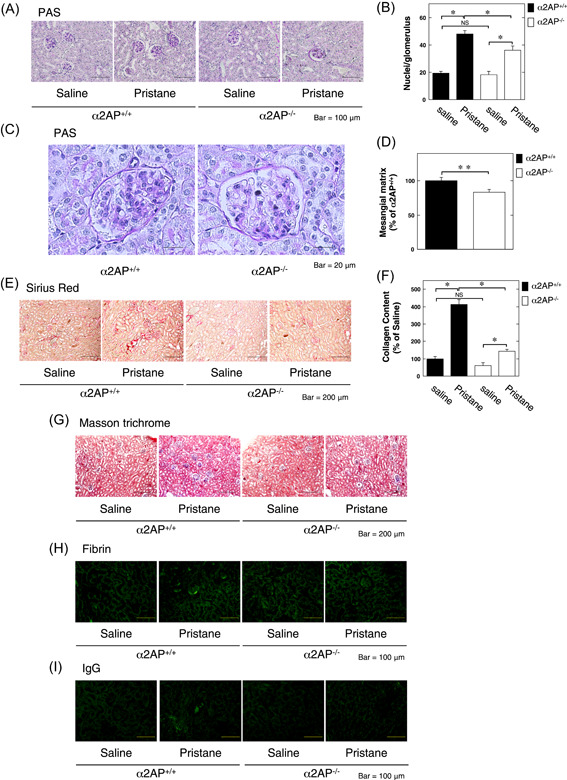
α2AP deficiency attenuated the development of lupus nephritis in the pristane‐induced lupus mouse model. A, Represetative kidney sections from saline or pristane‐treated α2AP^+/+^ and α2AP^−/−^ mice (periodic acid Schiff [PAS] stain). B, The number of nuclei per glomerulus in the saline or pristane‐treated α2AP^+/+^ and α2AP^−/−^ mice (n = 10). C, The magnified image of kidney sections from pristane‐treated α2AP^+/+^ and α2AP^−/−^ mice (PAS stain). D, Mesangial matrix was measured by PAS stain in the kidney from pristane‐treated α2AP^+/+^ and α2AP^−/−^ mice (n = 4). Mesangial matrix area was expressed as a percent of the observed with control in the pristane‐treated α2AP^+/+^ mice. E, Representative kidney sections from the saline or pristane‐treated α2AP^+/+^ and α2AP^−/−^ mice (sirius red stain). F, The collagen content in the kidney from saline or pristane‐treated α2AP^+/+^ and α2AP^−/−^ mice (n = 4). G, Represetative kidney sections from the saline or pristane‐treated α2AP^+/+^ and α2AP^−/−^ mice (masson trichrome stain). H, Paraffin sections of the kidney of the α2AP^+/+^ and α2AP^−/−^ mice were stained with anti‐fibrin antibodies. I, Paraffin sections of the kidney of the α2AP^+/+^ and α2AP^−/−^ mice were stained with immunoglobulin G antibodies. The data represent the mean ± SEM. *P < .01. **P < .05. α2AP, alpha2‐antiplasmin; NS, not significant

**Figure 3:**
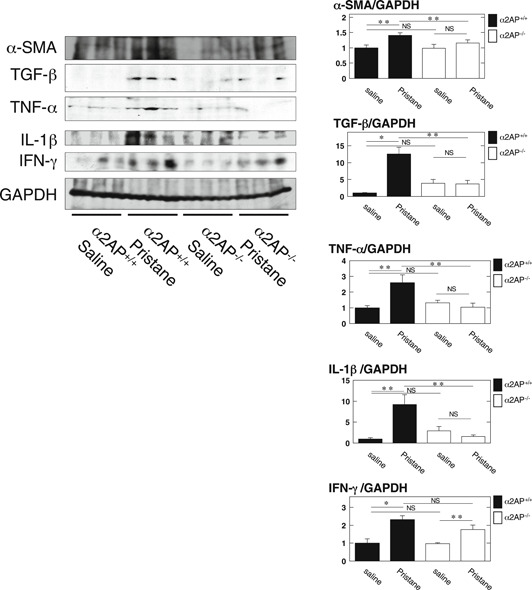
α2AP deficiency attenuated the inflammatory cytokine production in the pristane‐induced lupus mouse model. The expression of each protein in kidney from the saline or pristane‐treated α2AP^+/+^ and α2AP^−/−^ mice was examined by Western blot analysis. The histogram on the right panels shows quantitative representations of each protein obtained from densitometry analysis (n = 3). The data represent the mean ± SEM. *P < .01. **P < .05. α2AP, alpha2‐antiplasmin; NS, not significant

To clarify the effect of α2AP in the development of LN in the pristane‐induced lupus mouse model, we compared the pathologic changes in the kidney from pristane‐treated α2AP^+/+^ and α2AP^−/−^ mice. The administration of pristane‐induced glomerular cell proliferation (Figure 2A,B), mesangial matrix expansion (Figure 2C,D), and collagen production (Figures 2E‐G) in the kidney, and the levels of these products in pristane‐treated α2AP^+/+^ mice were significantly higher than those in pristane‐treated α2AP^−/−^ mice. In addition, fibrin deposition (Figure 2H) and IgG deposition (Figure 2I) were detected in the glomeruli of pristane‐treated α2AP^+/+^ mice, but these products were not detected in α2AP^−/−^ mice. However, the administration of pristane induced the infiltration of T‐cells and macrophages in the kidney, but there was no difference in the infiltration of T‐cells and macrophages in the pristane‐treated α2AP^+/+^ and α2AP^−/−^ mice (data not shown). In addition, the levels of anti‐dsDNA antibody production in the serum from pristane‐treated α2AP^+/+^ and α2AP^−/−^ mice were not induced (data not shown). Next, we showed that the expression of α‐smooth muscle actin (a hallmark of the myofibroblast phenotype), tumor necrosis factor‐α (TNF‐α), interleukin‐1β (IL‐1β), and transforming growth factor‐β (TGF‐β) in the kidney from pristane‐treated α2AP^+/+^ mice was significantly higher than that in the kidneys from pristane‐treated α2AP^−/−^ mice (Figure 3). However, there was no difference in the expression of interferon‐γ (IFN‐γ) in the kidney from the pristane‐treated α2AP^+/+^ and α2AP^−/−^ mice (Figure 3).

### 3.3 Interferon‐γ **induced the production of α2AP in fibroblasts**

**Figure 4:**
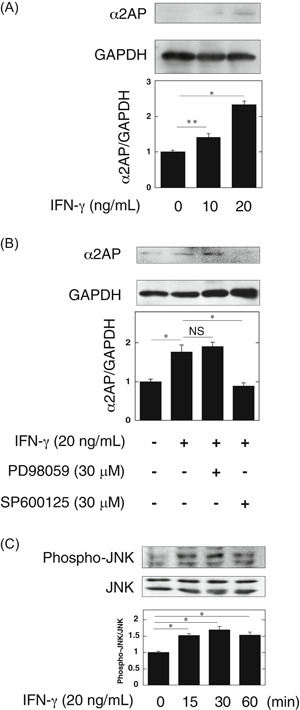
Interferon‐γ (IFN‐γ) induced the α2AP production through the c‐Jun N‐terminal kinase (JNK) pathway in fibroblasts. A, NIH3T3 cells were stimulated by IFN‐γ (10, 20 ng/mL) for 24 hours. The expression of α2AP was measured by Western blot analysis. The histogram on the bottom panels shows quantitative representations of α2AP expression obtained from densitometry analysis (n = 3). B, NIH3T3 cells were cultured for 24 hours in the absence or presence of IFN‐γ (20 ng/mL), PD98059 (30 μM), or SP600125 (30 μM) as indicated. The histogram on the bottom panels shows quantitative representations of α2AP expression obtained from densitometry analysis (n = 3). C, NIH3T3 cells were stimulated by IFN‐γ (20 ng/mL) for the indicated periods. The expression of each protein was measured by Western blot analysis. The histogram on the bottom panels shows quantitative representations of phospho‐JNK expression obtained from densitometry analysis (n = 3). The data represent the mean ± SEM. *P < .01. **P < .05. α2AP, alpha2‐antiplasmin; NS, not significant

α2AP deficiency attenuated the pristane‐induced LN progression and the production of proinflammatory cytokines, such as TNF‐α and IL‐1β. However, α2AP deficiency did not attenuate the pristane‐induced IFN‐γ production. These data suggest that IFN‐γ may be an upstream factor of α2AP‐mediated LN progression. Therefore, we focused on IFN‐γ, and found that IFN‐γ induced α2AP production in NIH3T3 fibroblasts (Figure 4A). In our previous study, we demonstrated that the activation of ERK1/2 or JNK pathways is associated with α2AP production.^27^ Therefore, we examined whether the ERK1/2 or JNK pathway is associated with the IFN‐γ‐induced production of α2AP by using the MEK specific inhibitors (PD98059) or JNK inhihitor (SP600125). SP600125 attenuated the IFN‐γ‐induced production of α2AP in NIH3T3 fibroblasts. (Figure 4B). We also confirmed that IFN‐γ activated the JNK pathway (Figure 4C).

### 3.4 The effect of plasmin on the IFN‐γ‐induced production of proinflammatory cytokine in macrophages

**Figure 5:**
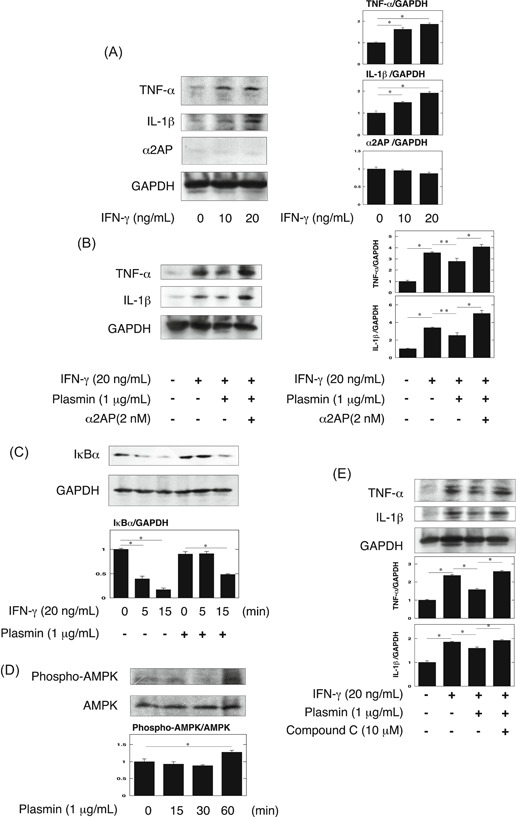
Plasmin attenuated the IFN‐γ‐induced inflammatory cytokine production through the AMPK pathway in macrophages. A, RAW264.7 cells were stimulated by IFN‐γ (10, 20 ng/mL) for 48 hours. The expression of tumor necrosis factor‐α (TNF‐α) and interleukin‐1β (IL‐1β) was measured by Western blot analysis. The histogram on the right panels shows quantitative representations of TNF‐α and IL‐1β expression obtained from densitometry analysis (n = 3). B, RAW264.7 cells were cultured for 48 hours in the absence or presence of IFN‐γ (20 ng/mL), plasmin (1 μg/mL), or α2AP (2 nM) as indicated. The histogram on the right panels shows quantitative representations of TNF‐α and IL‐1β expression obtained from densitometry analysis (n = 3). C, RAW264.7 cells were pretreated with 1 μg/mL plasmin for 30 minutes and then stimulated with 20 ng/mL IFN‐γ for the indicated periods. Degradation of IκBα was evaluated by a Western blot analysis. The histogram on the bottom panels shows quantitative representations of IκBα expression obtained from densitometry analysis (n = 3). D, RAW264.7 cells were stimulated by plasmin (1 μg/mL) for the indicated periods. The expression of phospho‐AMPK and AMPK was measured by Western blot analysis. The histogram on the bottom panel shows quantitative representations of phospho‐AMPK expression obtained from densitometry analysis (n = 3). E, RAW264.7 cells were cultured for 48 hours in the absence or presence of IFN‐γ (20 ng/mL), plasmin (1 μg/mL), or compound C (10 μM) as indicated. The histogram on the bottom panels shows quantitative representations of TNF‐α and IL‐1β expression obtained from densitometry analysis (n = 3). The data represent the mean ± SEM. *P < .01. **P < .05. α2AP, alpha2‐antiplasmin; IFN‐γ, interferon‐γ

IFN‐γ is known to promote innate immune and inflammatory responses, and be essential for the induction of LN.^28^, ^29^, ^30^ In addition, macrophages reportedly contribute to LN progression by secreting proinflammatory cytokines including TNF‐α and IL‐1β.^31^ Therefore, we examined that the effect of IFN‐γ and plasmin/α2AP in the production of proinflammatory cytokine in macrophages. As shown in Figure 5A, IFN‐γ induced the production of proinflammatory cytokines, such as TNF‐α and IL‐1β in RAW264.7 macrophages. The activation of AMPK is known to inhibit nuclear factor‐κB activation and IFN‐γ signaling,^32^, ^33^, ^34^ and we previously showed that plasmin activates the AMPK pathway, and the AMPK activation attenuated inflammation response.^11^, ^35^ Therefore, we examined the possible involvement of plasmin in the IFN‐γ‐induced TNF‐α and IL‐1β production. Plasmin attenuated the IFN‐γ‐induced TNF‐α and IL‐1β production, and the treatment of α2AP eliminated the plasmin‐attenuated effects in RAW264.7 macrophages (Figure 5B). In addition, plasmin attenuated the IFN‐γ‐induced IκBα degradation in RAW264.7 macrophages (Figure 5C). Furthermore, plasmin activated the AMPK pathway (Figure 5D), and the AMPK inhibitor compound C^36^ eliminated the plasmin‐attenuated effects in RAW264.7 macrophages (Figure 5E).

### 3.5 The effect of α2AP on the production of proinflammatory cytokine in macrophages

**Figure 6:**
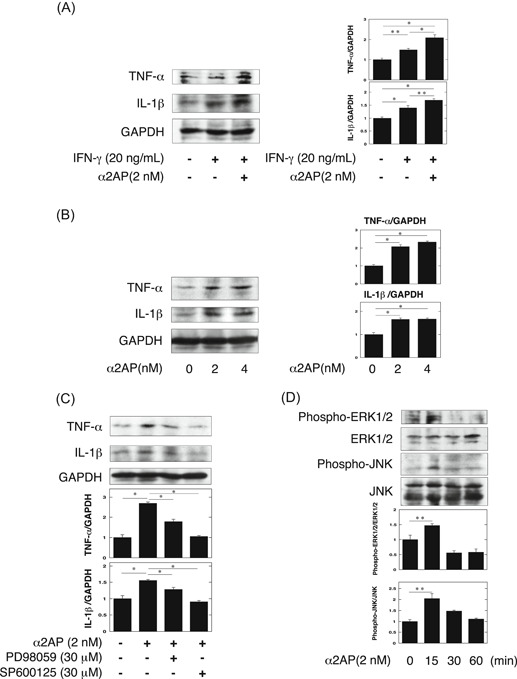
α2AP induced the inflammatory cytokines through the ERK1/2 and JNK pathway in macrophages. A, RAW264.7 cells were cultured for 48 hours in the absence or presence of IFN‐γ (20 ng/mL) or α2AP (2 nM) as indicated. The histogram on the right panels shows quantitative representations of TNF‐α and IL‐1β expression obtained from densitometry analysis (n = 3). B, RAW264.7 cells were stimulated by α2AP (2, 4 nM) for 48 hours. The expression of TNF‐α and IL‐1β was measured by Western blot analysis. The histogram on the right panels shows quantitative representations of TNF‐α and IL‐1β expression obtained from densitometry analysis (n = 3). C, RAW264.7 cells were cultured for 48 hours in the absence or presence of α2AP (2 nM), PD98059 (30 μM), or SP600125 (30 μM) as indicated. The histogram on the bottom panels shows quantitative representations of TNF‐α and IL‐1β expression obtained from densitometry analysis (n = 3). D, RAW264.7 cells were stimulated by α2AP (2 nM) for the indicated periods. The expression of each protein was measured by Western blot analysis. The histogram on the bottom panels shows quantitative representations of phospho‐ERK1/2 and phospho‐JNK expression obtained from densitometry analysis (n = 3). The data represent the mean ± SEM. *P < .01. **P < .05. α2AP, alpha2‐antiplasmin; JNK, c‐Jun N‐terminal kinase; IFN‐γ, interferon‐γ; IL‐1β, interleukin‐1β; TNF‐α, tumor necrosis factor‐α

Next, we showed that IFN‐γ induced the production of proinflammatory cytokines, such as TNF‐α and IL‐1β in RAW264.7 macrophages, and α2AP promoted them in the absence of plasmin (Figure 6A). It has been reported that α2AP is associated with the production of several cytokines.^12^, ^18^ Therefore, we examined the effect of α2AP in the production of TNF‐α and IL‐1β in macrophages, and showed that the treatment of α2AP induced the production of TNF‐α and IL‐1β in RAW 264.7 macrophages (Figure 6B). In addition, we examined whether the ERK1/2 or JNK pathway is associated with the α2AP‐induced effects by using the MEK specific inhibitors (PD98059) or JNK inhihitor (SP600125). PD98059 and SP600125 attenuated the α2AP‐induced effects in RAW264.7 macrophages. (Figure 6C). We also confirmed that α2AP activated ERK1/2 and JNK pathways (Figure 6D).

## 4 DISCUSSION

In the present study, we examined the role of α2AP in the pathogenesis of LN using the pristane‐induced lupus mouse model and showed that the levels of PAP and α2AP were elevated in this model (Figure 1). In addition, we showed that α2AP deficiency attenuated glomerular cell proliferation, mesangial matrix expansion, collagen production, fibrin deposition, IgG deposition, and proinflammatory cytokine production (Figures 2 and 3). Immune deposition, inflammatory responses, hypercoagulability, and thrombosis are associated with LN progression.^5^, ^6^, ^7^, ^8^ α2AP not only regulates PAP formation and fibrin deposition through plasmin binding and inhibition but is also associated with the production of several cytokines.^12^, ^18^ PAP reportedly induces the production of IgG and IgM in mononuclear cells,^37^ and α2AP is associated with the production of IgE and the recruitment of immune cells.^19^, ^38^, ^39^ In addition, fibrin accumulation is known to induce the inflammatory response through multiple mechanisms.^32^, ^33^ Furthermore, we demonstrated in the present study that α2AP induced proinflammatory cytokine production in macrophages (Figure 6). Increased α2AP expression may induce plasmin inhibition, impair fibrinolysis and proinflammatory cytokine production, and contribute to immune deposition, inflammatory responses, hypercoagulability, and thrombosis. However, in the present study, the anti‐dsDNA antibody production was not induced in the serum from pristane‐treated α2AP^+/+^ and α2AP^−/−^ mice (data not shown). It is probable that anti‐dsDNA antibody production was not induced because the period of pristane administration in this study was short. The long‐term pristane administration may further induce α2AP production and exacerbate LN progression. Further investigations would be required to clarify the details.

In the present study, we showed that α2AP deficiency attenuated TNF‐α and IL‐1β production but did not attenuate the IFN‐γ production in these mice (Figure 3). These findings indicate that IFN‐γ, which is known to be essential for the induction of LN, may be an upstream factor of α2AP. Therefore, we examined whether IFN‐γ is associated with the increase in α2AP expression, and found that IFN‐γ induced α2AP production through the JNK pathway in fibroblasts (Figure 4). Next, macrophages reportedly contribute to LN progression by secreting proinflammatory cytokines including TNF‐α and IL‐1β.^31^ Therefore, we examined that the effect of IFN‐γ and plasmin/α2AP in the proinflammatory cytokine production in macrophages, and showed that IFN‐γ induced proinflammatory cytokine production in macrophages, plasmin attenuated the IFN‐γ‐induced proinflammatory cytokine production through the AMPK pathway, and α2AP eliminated these effects (Figure 5). Plasmin also reportedly inhibits TNF‐α‐induced apoptosis in monocytes,^34^ regulates the clearance of dead cells,^40^ and plays a role in the pathogenesis of glomerulonephritis.^41^ Furthermore, we showed that α2AP induced proinflammatory cytokine production through the ERK and JNK pathways in macrophages (Figure 6). The increase in α2AP may therefore not only induce proinflammatory cytokine production but also inhibit the plasmin function, and exacerbate the disease severity. These findings suggest that α2AP is associated with the immune and inflammatory responses in the induction and development of LN through plasmin inhibition and α2AP itself functions.

Severe LN is characterized by inflammatory glomerular lesions resulting in fibrosis and a loss of the renal function.^42^ The chronic inflammation response is known to cause the development of fibrosis.^43^ Fibrosis is characterized by the deposition of myofibroblasts and extracellular matrix. We showed that profibrotic responses, such as myofibroblast differentiation and collagen deposition and profibrotic cytokine TGF‐β production were induced in the lupus model mice, and α2AP deficiency attenuated these effects (Figure 2). We previously showed that α2AP induced myofibroblast differentiation and collagen deposition and was associated with the development of fibrosis.^13^, ^14^, ^15^, ^27^ In contrast, plasmin is known to exert to antifibrosis effects through the activation of matrix metalloproteinase^44^ and hepatocyte growth factor^45^ and the promotion of myofibroblast apoptosis.^46^ A chronic increase in the α2AP expression may promote the development of LN and exacerbate fibrosis. Furthermore, it has been reported that the levels of α2AP are also elevated in patients with rheumatic diseases other than SLE,^47^, ^48^, ^49^, ^50^ and the blockade of α2AP attenuates the disease severity in systemic sclerosis (SSc) model mice and SSc fibroblasts.^47^, ^51^ α2AP might therefore play an important role in the initiation and progression of various rheumatic diseases, suggesting that its blockade might be useful for treating various rheumatic diseases.

In conclusion, α2AP is associated with the development of LN through the regulation of plasmin inhibition and the inflammatory responses. Our findings may provide new insight into the development of clinical therapies for LN.

## CONFLICT OF INTERESTS

The authors declare that there are no conflict of interests.

## AUTHOR CONTRIBUTIONS

YK conceived and designed the experiment. YK and MM were involved in the mice experiments. YK and MM analyzed the data. YK, MS, and OM were involved in data interpretation and writing of the manuscript.
